# Cigarette Smoking Aggravates the Activity of Periodontal Disease by Disrupting Redox Homeostasis- An Observational Study

**DOI:** 10.1038/s41598-018-29163-6

**Published:** 2018-07-23

**Authors:** Chia-Huang Chang, Ming-Lun Han, Nai-Chia Teng, Chang-Yu Lee, Wan-Ting Huang, Che-Tong Lin, Yung-Kai Huang

**Affiliations:** 1 College of Public Health and Nutrition, Taipei Medical University, Taipei, 110 Taiwan; 2 Department of Internal Medicine, Min-Sheng General Hospital, Taoyuan, 330 Taiwan; 3 School of Dentistry, College of Oral Medicine, Taipei Medical University, Taipei, 11s0 Taiwan; 4 Division of Periodontics, Department of Dentistry, Taipei Medical University Hospital, Taipei Medical University, Taipei, 110 Taiwan; 5 School of Oral Hygiene, College of Oral Medicine, Taipei Medical University, Taipei, 110 Taiwan

## Abstract

The aim of this study was to evaluate the associations between cigarette use and five salivary oxidative stress biomarkers, copper-zinc superoxide dismutase (Cu/Zn SOD), manganese superoxide dismutase (MnSOD), catalase, thioredoxin-1 (TRX1), and peroxiredoxin-2 (PRX2), to assess the effectiveness of non-surgical periodontal therapy. Materials and Methods: This is an observational study,167 patients diagnosed with periodontitis were recruited. Both saliva samples and clinical measurements (plaque index (PI), bleeding on probing (BOP), and pocket depth (PD)) were taken at baseline and after completing non-surgical periodontal therapy. The Levels of salivary biomarkers were determined using a MILLIPLEX^®^ MAP Human Oxidative Stress Magnetic Bead Panel kit. The overall reductions in PI and BOP were 31.56% and 42.16%, respectively. BOP reduction after treatment in female or male non-smokers was significantly higher than in male former smokers (p < 0.05). After completing non-surgical periodontal therapy, Cu/ZnSOD, MnSOD, catalase, and Prx2 significantly decreased. There was a significant interaction between smoking status and ΔCu/ZnSOD on PI and a significant interaction between smoking status and ΔCatalase on BOP. Conclusions: Cigarette smoking interferes with redox homeostasis in the body, alters antioxidants levels, and influences the periodontal disease activity.

## Introduction

Periodontal disease is one of the most common chronic diseases, with a gradually increasing prevalence worldwide^1^. The United States National Health and Nutrition Examination Survey (NHANES) showed that the periodontal disease prevalence in adults age 30 years or older decreased from 47.2% (NHANES 2009–2010) to 44.8% (NHANES 2011–2012)^2,3^. In Taiwan, it was estimated that approximately 54% of adults aged 35–44 years had mild to severe periodontitis^4^. The prevalence of periodontal disease has significantly increased in Taiwan over the past 17 years^5^. Periodontal disease induces a series of immune inflammatory responses that lead to the destruction of periodontal tissues and even tooth loss^6^. Clinical evidence indicates that cardiovascular diseases, diabetes mellitus, and other chronic diseases may be associated with periodontal inflammation^1^.

Reactive oxygen species (ROS), such as superoxide and hydroxyl species, are regulated by the thioredoxin (TRX) and peroxiredoxin (PRX) systems to transduce redox signals and alter activities of antioxidant enzymes to eliminate free radicals^7^. When ROS are generated, the TRX system is stimulated and transduces redox signals to alter the activities of antioxidant enzymes, such as superoxide dismutase (SOD), catalase, and glutathione peroxidase (GPx), to eliminate free radicals. Dismutation of the superoxide radical (O2•−), which is released from ROS, is catalyzed by SOD, which transforms it into hydrogen peroxide (H_2_O_2_). H_2_O_2_ is scavenged by catalase, peroxiredoxin-2 (Prx2), and glutathione, by transforms it into H_2_O and O_2_^8^. Those antioxidants are induced when oxidative stress is relieved. Salivary oxidative biomarkers, such as the total oxidant status and SOD, have been useful in evaluating the severity of periodontal disease^9^. The association between the salivary oxidative status and periodontal disease in previous studies is conflicting^10–12^. The roles oxidative stress biomarkers play in the entire process of generating ROS in periodontitis need to be investigated.

Cigarette smoking is one of the most important risk factors for periodontal disease^1^. In the USA, 41.9% of adult periodontitis cases are attributable to current cigarette smoking and 10.9% to former smoking^13^. In the USA, the prevalence of periodontal disease is approximately 40%, and current smokers have at least a 50% greater likelihood of periodontitis in comparison with non-smokers^14^. Epidemiological studies have shown that smokers have less improvement than nonsmokers following non-surgical periodontal treatment^15^. Exposure to cigarette smoke increases the levels of ROS, which are harmful to the human body due to their disruption of redox homeostasis^16–18^. Cigarette smoking may interfere with the oxidative stress mechanisms of periodontal tissues, inhibit defenses against plaque bacteria, and lead to vascular constriction and slow wound healing^19^. Research on oxidative stress has largely focused on the role and effect of oxidative stress in protecting these molecules from damage. However, associations between salivary oxidative stress, the smoking status, and the effectiveness of periodontal treatment are rarely mentioned.

The goal of periodontal treatment is to achieve effective infection control, recover periodontal function, and maintain the esthetics of dentition^20^. However, inferior treatment responses related to the redox homeostasis and individual susceptibility are seldom reported. Our hypothesis was that smoking affects periodontal treatment through induction of oxidative stress. The aim of the present study was to evaluate the associations between cigarette use and salivary oxidative stress biomarkers in subjects undergoing non-surgical periodontal therapy.

## Results

Demographic characteristics stratified by the smoking status of the study subjects are described in Table 1. The median ages were 55.24, 59.48, 61.19, and 50.25 years among non-smoking females, non-smoking males, male former smokers, and male current smokers, respectively (p = 0.03, Kruskal-Wallis test). The age of male former smokers was significantly higher than those in male current smokers after Bonferroni post hot test. Current smokers had high rates of alcohol consumption and exposure to environmental tobacco smoke. In a further comparison of smoking habits between former and current smokers, there were no statistically significant differences regarding the smoking quantity, duration, or pack-years and no statistically significant differences existed for the educational level, marital status, or salivary properties between these four groups (Table S1).

**Table 1:** Baseline characteristics of non-smokers, former smokers, and current smokers.

	Female	Male			*p* value
	Non-smoker	Non-smoker	Former smoker	Current smoker	
Total subject Number	88	39	21	19	
Annual income (NTD), N (%)					
<500,000	61 (69.32)	20 (51.28)	9 (42.86)	5 (26.32)	0.01^a^
500,000~1,000,000	16 (18.18)	7 (17.95)	5 (23.81)	7 (36.84)	
>1,000,000	11 (12.50)	12 (30.77)	7 (33.33)	7 (36.84)	
Alcohol consumption, N (%)					
Never	81 (92.05)	25 (64.10)	6 (28.57)	11 (57.89)	<0.01^a^
Former/current	7 (7.95)	14 (35.90)	15 (71.43)	8 (42.11)	
Environmental tobacco smoke, N (%)					
No	70 (79.55)	30 (76.92)	19 (90.48)	10 (52.63)	0.04^a^
Yes	18 (20.45)	9 (23.08)	2 (9.52)	9 (47.37)	
Age, median (QD)	55.24 (3.58)	59.48 (10.55)	61.19 (6.02)	50.25 (5.90)	0.03^b^

QD, quartile deviation. NTD, New Taiwan dollars (in 2016, the average exchange rate was US$1.00 ≈ NTD30). ^a^p value for Kruskal-Wallis test. ^b^p value for Wilcoxon rank-sum test.

**Figure 1:**
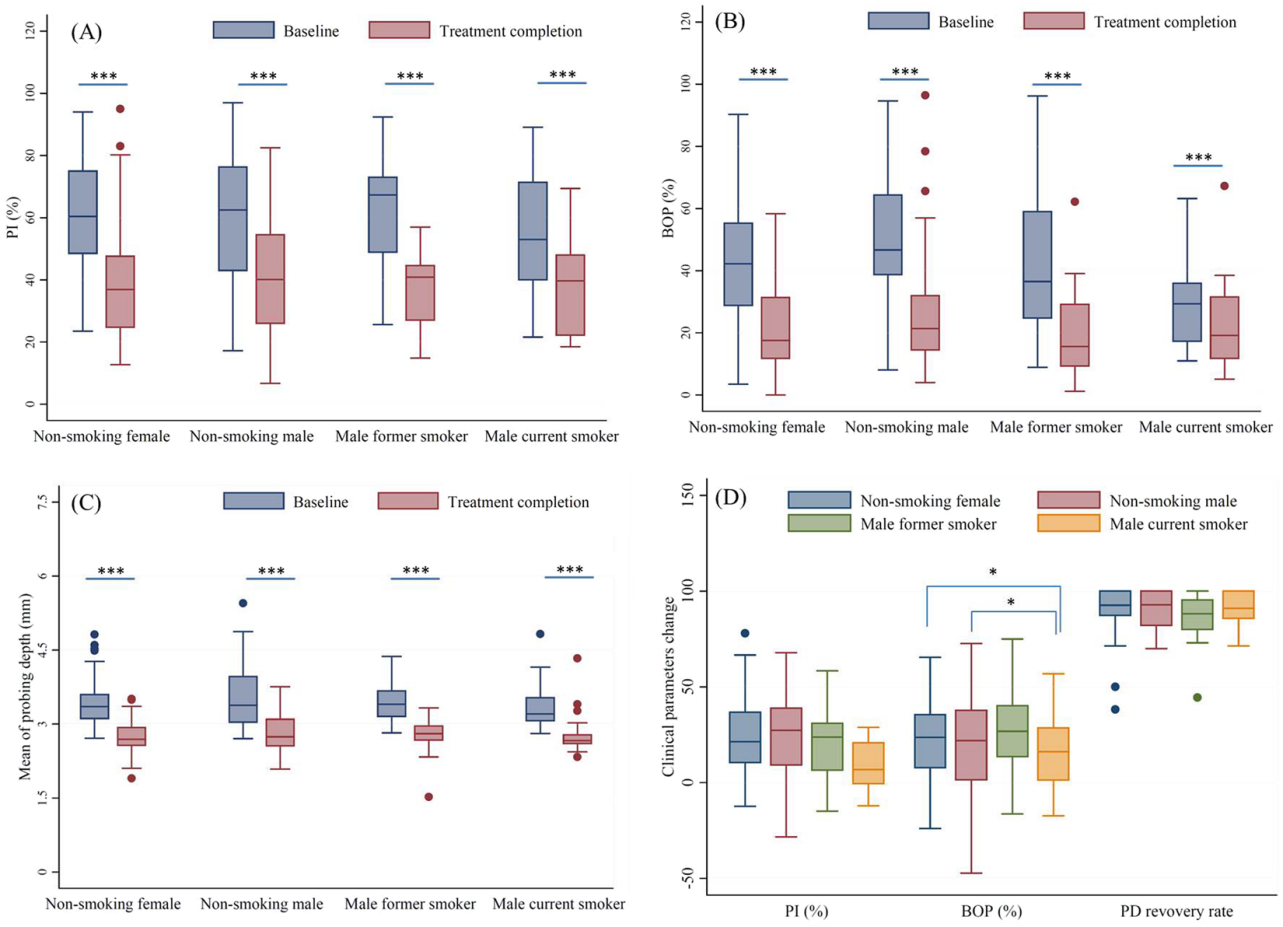
Periodontal parameters classified by smoking status at baseline and treatment completion. ***p value < 0.0001, Wilcoxon signed-rank test. *p value < 0.05, Kruskal-Wallis test and Bonferroni post hoc test. (**A**) PI; (**B**) BOP; (**C**) mean probing depth; (**D**) reduction in clinical parameters strata by smoking status.

Figure 1 shows the clinical parameters of subjects at baseline and after treatment stratified by gender and smoking status. The before and after treatment data (median, first quartile and third quartile) of clinical parameters are listed in Table S2. Baseline BOP in male smokers was significantly lower than those in female or male non-smokers. Baseline PI, PD mean or after treatment clinical parameters were similar between those groups which stratified by gender and smoking status. All of the clinical parameters were significantly lower than those at baseline in each stratum of gender and smoking status (Fig. 1A to C, p < 0.0001). To evaluate the association between smoking effect and clinical treatment efficiency, the reduction of clinical parameters from baseline and after completing treatment were showed in Fig. 1D. The reduction of PI or BOP was calculated by subtracting the PI or BOP at after completing treatment from that at baseline. The PD recovery rate was defined as an above 2 mm improvement of pocket depth for east one periodontal pocket of 5–9 mm on each side of the mouth. All the reduction data (median, first quartile and third quartile) of clinical parameters are listed in Table S3. BOP reduction in male smokers was significantly lower than those in female or male smokers (p < 0.05). The reduction of PI, PD mean and PD recovery rate were similar between female non-smokers, male non-smokers, male former smokers, and male current smokers (Fig. 1D).

**Figure 2:**
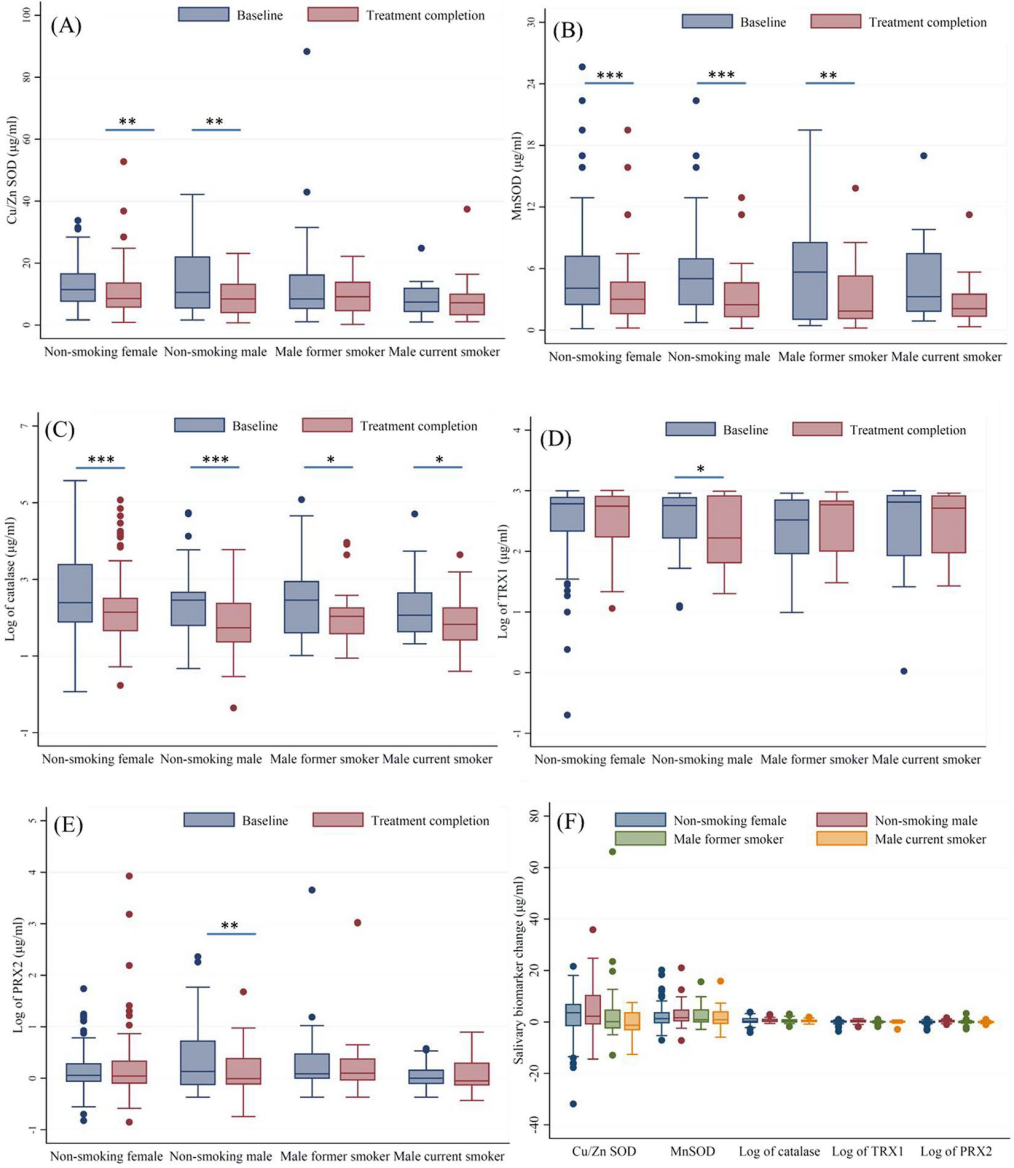
Salivary antioxidant biomarker levels at baseline and treatment completion. (**A**) Cu/ZnSOD; (**B**) MnSOD; (**C**) Log of catalase; (**D**) Log of TRX1; (**E**) Log of PRX2; (**F**) reduction in antioxidant biomarker strata by smoking status. *p value < 0.05, **p value < 0.001, ***p value < 0.0001 by Wilcoxon signed-rank test.

Figure 2 shows the salivary oxidative stress biomarker levels at baseline and after completing treatment by gender and smoking status. For non-smoking males, all of five salivary markers were significantly decreased after treatment completion. The before and after treatment data (median, first quartile and third quartile) of five salivary markers are listed in Table S4. Similar decreases for Cu/ZnSOD, MnSOD, and catalase were observed among non-smoking females. For former smokers, the median MnSOD and catalase levels significantly decreased from 5.67 to 1.86 µg/mL (*p* < 0.01) and 287.13 to 108.51 µg/mL (*p* = 0.03), respectively. For current smokers, only catalase was significantly increased after treatment: the median dropped from 116.22 to 67.1 µg/mL (*p* = 0.01). No significant differences in the Trx1 level between baseline and after treatment were found in the 4 groups. The reductions of five salivary biomarkers were similar between female non-smokers, male non-smokers, male former smokers, and male current smokers (Fig. 2F).

Table 2 shows Spearman’s correlation coefficients between the periodontal parameters and five biomarkers of oxidative stress. At baseline, the Cu/ZnSOD, MnSOD, and catalase levels were significantly associated with the PI (*r* = 0.29–0.30, *p* < 0.01) and BOP (*r* = 0.31–0.42, *p* < 0.01). After completion of treatment, a significant correlation only existed between BOP and catalase (*r* = 0.22, *p* < 0.01).

**Table 2:** Spearman correlation coefficients between the periodontal parameters and antioxidants at baseline and after completing treatment.

Baseline	PI (%)	BOP (%)	PD recovery (%)
Cu/ZnSOD (µg/ml)	0.30**	0.31**	0.05
MnSOD (µg/ml)	0.29**	0.39**	0.15
Catalase (µg/ml)	0.29**	0.42**	0.14
Trx1 (µg/ml)	0.12	−0.10	0.03
Prx2 (µg/ml)	−0.04	−0.03	0.05
**After completing treatment**	**PI (%)**	**BOP (%)**	**PD recovery (%)**
Cu/ZnSOD (µg/ml)	0.14	0.14	−0.10
MnSOD (µg/ml)	0.02	0.15	0.04
Catalase (µg/ml)	0.08	0.22**	0.01
Trx1 (µg/ml)	−0.06	0.05	0.01
Prx2 (µg/ml)	0.12	0.04	−0.08

*p < 0.05; **p < 0.01.

PI, plaque index; BOP, bleeding on probing; PD, pocket depth; Cu/ZnSOD, copper-zinc superoxide dismutase; MnSOD, manganese superoxide dismutase; CAT, catalase; Trx1, thioredoxin-1; Prx2, peroxiredoxin-2.

Repeated-measures ANOVA was used to calculate the adjusted changes in PI, BOP and PD mean for the combined effect of smoking status and the differences in salivary oxidative stress marker levels (Table 3). Smoking status had a significant effect on BOP (p = 0.01–0.04), but the effect was not found in the PI or PD mean. Salivary ΔCu/Zn SOD had a significant effect on PI (p = 0.03) and BOP (p = 0.04). Salivary ΔMnSOD had a significant effect on PD mean (p = 0.04). Salivary ΔCatalase had a significant effect on BOP (p < 0.001). There was a significant interaction between smoking status and ΔCu/Zn SOD on PI (p = 0.03). The change of PI with time in male non-smoker who had ΔCu/Zn SOD < 0 µg/ml were significantly lower than those in male non-smoker who had ΔCu/Zn SOD > 5.4 µg/ml (PI change mean valve: 2.24% v.s. 32.94%, respectively; Bonferroni correction adjusted p value was 0.03). Although there was no significant effect of smoking status or ΔTrx1 on PI (p = 0.30 and 0.69), there was a significant interaction between smoking status and ΔTrx1 on PI (p = 0.04). There was no any significantly difference across subgroup of smoking status and ΔTrx1 strata. Finally, there was a significant interaction between smoking status and ΔCatalase on BOP. The change of BOP with time in female non-smoker who had ΔCatalase > 225 µg/ml were significantly higher than those in male non-smoker or male smoker who had ΔCatalase 0–255 µg/ml (BOP change mean valve: 32.12% v.s. 13.11% or 8.87%, respectively; Bonferroni correction adjusted p value was <0.01 and 0.04, respectively). The change of BOP with time in male non-smoker who had ΔCatalase >225 µg/ml were significantly higher those in male non-smoker who had ΔCatalase 0–255 µg/ml or male former smoker who had Δcatalase >225 µg/ml or male smoker who had ΔCatalase 0–255 µg/ml or had Δcatalase >255 µg/ml (BOP change mean valve: 32.12% v.s. 13.11% or 12.37% or 8.87% or 5.17%, respectively; Bonferroni correction adjusted p value was <0.001, 0.03, <0.01 and <0.01, respectively).

**Table 3:** Results of two-way repeated-measures ANOVA comparing the main effects of smoking status and oxidative stress markers on the clinical parameters.

Dependent variable	PI (%)		BOP (%)		PD mean (mm)	
Independent variable	F value	P value	F value	P value	F value	P value
Smoking status effect	1.50	0.22	2.68	0.04	2.09	0.10
ΔCu/Zn SOD effect	3.76	0.03	3.38	0.04	0.36	0.70
Smoking status × ΔCu/Zn SOD effect	2.40	0.03	1.03	0.41	1.26	0.28
Smoking status effect	0.53	0.66	3.49	0.02	1.16	0.33
ΔMnSOD effect	1.11	0.33	1.36	0.26	3.3	0.04
Smoking status × ΔMnSOD effect	1.44	0.20	0.34	0.92	0.32	0.93
Smoking status effect	0.87	0.46	3.86	0.01	0.59	0.62
ΔCatalase effect	4.55	0.01	5.7	<0.001	1.61	0.20
Smoking status × ΔCatalase effect	0.43	0.86	2.59	0.02	1.23	0.29
Smoking status effect	1.22	0.30	3.16	0.03	1.24	0.30
ΔTrx1 effect	0.38	0.69	0.82	0.44	0.28	0.75
Smoking status × ΔTrx1 effect	2.26	0.04	0.42	0.87	0.31	0.93
Smoking status effect	0.79	0.50	2.69	0.04	0.94	0.42
ΔPrx2 effect	0.27	0.77	1.15	0.32	0.06	0.94
Smoking status × ΔPrx2 effect	0.36	0.90	0.79	0.58	0.19	0.98

PI, plaque index; BOP, bleeding on probing; PD, pocket depth; SOD1, copper-zinc superoxide dismutase; SOD2, manganese superoxide dismutase; CAT, catalase; TRX1, thioredoxin-1; PRX2, peroxiredoxin-2.

## Discussion

In this study, the majority of participants exhibited good responses to non-surgical periodontal treatments. The overall reductions in PI and BOP were 31.56% and 42.16%, respectively. Compared to other studies, the reductions in PI and BOP in this study were clinically acceptable^21–23^. Approximately 20% of patients still had increases in PI and BOP after treatment, and no significant difference regarding the smoking status of these patients existed. The outcomes of non-surgical periodontal therapy may be influenced by many factors. Cigarette smoking has *adverse effects* on periodontal treatments^24–26^. Preshaw *et al*. indicated that non-smokers tended to have less advanced periodontitis at baseline and better responses to non-surgical periodontal treatment^27^. Smoking is an important risk factor for periodontitis and other factors that influence the PI and BOP. The percentage of patients who required further periodontitis treatment was higher for smokers (42.8%) than for non-smokers (11.5%) after periodontal therapy^28^. The risk of treatment failure in smokers was 3.8 times that of non-smokers. BOP is an important index used to evaluate infection control during periodontitis treatment and reflects the inflammatory response when the gingiva is exposed to bacterial pathogens and the severity of inflammation^21,23^. In this study, current smokers had a lower percentage reduction in BOP compared to non-smokers and smoking status had a significant effect on BOP reduction.

In healthy gingiva, cigarette smoking acute exposure induced vascular constriction and gingival hyperaemia via systemic blood pressure increase^29^. But in the periodontitis patients, the BOP in smoker was significantly lower than non-smoker^30^. In our study, BOP in smoker was lower than those in the non-smoker. Effect on cigarette smoking in the healthy gingiva was different from periodontitis gingiva, and this difference may interference by the periodontal pathogen. As well as the periodontal pathogen, cigarette smoking also induced the inflammatory effect. Burning or heating of tobacco generates thousands of chemicals, and cigarette smoke modulates inflammation and promotes chronic inflammation by a variety of mechanisms^18^. Periodontal pathogen infection causes inflammation and oxidative stress and leads to the destruction of periodontal tissues^31,32^. Studies have also revealed that a *P. gingivalis* challenge can enhance pro-inflammatory factor production in human gingival human gingival fibroblasts and periodontal ligament fibroblasts^33^. Non-surgical periodontal treatments, such as scaling and oral hygiene instructions (including smoking cessation counseling), can eliminate periodontal pathogens and inflammation^34,35^. Post-operative BOP % were similar between those groups which stratified by gender and smoking status (Table S2) in this study, a significant interaction between smoking status and salivary catalase in BOP reduction was shown in this study. Cigarette smoking interferes with redox homeostasis and influences the periodontal disease activity.

Oxidative stress is an imbalance between the production of ROS and removal of reactive intermediates, including hydrogen peroxide, nitric oxide, superoxide, and other hydroxyl radicals. Specific oxidative stress biomarkers, such as SOD, reflect the severity of disease and effectiveness of periodontal treatment^9^. A low total antioxidant status has been reported in patients with periodontal disease due to the damaging effects of ROS^36^. A similar study indicated that the total antioxidant capacity was lower in patients with periodontitis than in healthy subjects^37^. Salivary total antioxidants and SOD remained unchanged in the control group (without periodontal therapy) of another study^38^. The SOD level has been correlated with many inflammatory diseases and could reflect the onset of disease^39^. Wei *et al*. found that the salivary SOD levels of chronic periodontitis patients significantly decreased after treatment (from 216.4 ± 36.8 to 169.8 ± 23.7 U/mg protein, *p* < 0.05)^12^, and a similar phenomenon was found for serum SOD. Novakovic *et al*. found that patients with periodontitis had higher SOD compared to periodontally healthy subjects and that the salivary SOD level significantly decreased after non-surgical treatment (from 0.45 ± 0.12 to 0.39 ± 0.23 IU/L, *p* < 0.01)^38^. The results of this study are consistent with the studies mentioned above; salivary SOD was associated with the PI and BOP at baseline and decreased after treatment. In addition, smokers had lower reductions in SOD compared to former smokers and non-smokers. An increase in SOD activity may be accompanied by an early inflammatory syndrome, while its alleviation occurs in response to pathological progression.

The influences of cigarette smoking on these five salivary oxidative biomarkers were not apparent in this study. There were no significant differences in the salivary antioxidant biomarker levels among non-smokers and former and current smokers at baseline or after completing treatment. A similar phenomenon was also indicated in other studies, which found that cigarette smoking did not influence the activities of antioxidant enzymes in humans^40–42^. However, it is worth noting that changes in oxidative stress markers after periodontal treatment in smoking subjects did occur. In this study, a significant interaction between smoking status and ΔCu/Zn SOD or ΔTrx1 at baseline and after treatment PI was shown. This finding implies that cigarette smoking interferes with redox homeostasis in the body, alters antioxidant levels in balancing ROS, and influences the periodontal disease activity.

Effective plaque control is the most important step in preventing dental caries and periodontal diseases^43^. The risk factors for periodontal disease, such as age, gender, educational level, an unhealthy diet, tobacco use, alcohol use, and dental care, have been examined in epidemiological studies^1^. The percentage of plaque present is a sensitive index that varies with food intake and oral hygiene. In an animal model, the dental plaque level decreased when rats were treated with an antioxidant-containing diet^44^. SOD defenses against oxidative stress which was caused by ROS accumulation. SOD is an oxidative stress biomarker, and higher SOD indicates higher oxidative stress. In this study, PI reduction was significantly accompanied by decreased SOD in non-smoking males and females. In non-smokers, having good oral hygiene (a lower plaque index) can decrease salivary oxidative stress.

Biofluids, such as serum, saliva, and gingival crevicular fluid, have been proposed to be good sources for prognostic biomarkers for periodontal tissue conditions, oxidant status, and therapy outcomes^12,38,45^. Accumulating evidence shows that the inflammatory mediators in saliva could be a biomarker for oral diseases, including oral cancer and periodontal disease. The association between biomarkers in gingival crevicular fluid (GSF) and periodontitis is less relevant than the association between salivary biomarkers and periodontitis (p = 0.04 vs. p < 0.001)^46^. The quality and quantity of publications on GSF are lower than for the other types of specimens^47^. Saliva samples, which contain unique information on oral physiological changes, can be useful diagnostic tools for periodontal diseases^48^. Saliva not only represents systemic circulation but is also easier to access and more widely applicable.

In this study, 57.5% of smokers had an alcohol consumption habit. Animal studies showed that alcohol consumption reduced plasma antioxidant activities (such as those of Cu/ZnSOD, MnSOD, CAT, and glutathione peroxidase) in a dose-dependent manner. On the other hand, alcohol-induced oxidative stress may be attributed to the promotion of membrane lipid peroxidation^49^. Epidemiological evidence reveals that alcohol consumption leads to overproduction of ROS and a decrease in anti-oxidative defense^50^. In this study, alcohol consumption status had no effect on the change of clinical parameters or salivary oxidative stress markers. The interaction between alcohol consumption and salivary oxidative stress markers was also not found in the clinical parameters changes. The interaction between smoking and alcohol in oxidative stress and periodontal disease activity needs to be investigated further.

Only one periodontist performed the examinations in this study, so the effects of therapy might be biased by reliability. This periodontist had more than ten years of clinical experience in periodontal disease treatment and standard periodontal diagnostic criteria, so the diagnosis and clinical evaluation in the present study should be reliable. This is a single study and has limited statistical power for salivary Trx1 and Prx2 analyses, so the precision of our estimates is threatened by the small sample. Because of the limited sample size, we should interpret our results (interaction between smoking status and ΔTrx1 on PI) with caution. In spite of the significant associations between oxidative stress markers and periodontal disease, the major limitation of this study was that the levels of oxidative stress are also influenced by other environmental factors and nutrition intake. To reduce inter-subject variability from the salivary biomarker estimates of the treatment effect, all of the salivary biomarkers were compared within the same subject in this study. Although this study showed an interaction between catalase and smoking status in BOP% reduction, female smokers were not included in this study, and the results of this study may not be able to be generalized or extrapolated to populations of female smokers. Smoking cessation and consumption of antioxidant-rich foods or potent antioxidant supplements during periodontal treatment could have reduced inflammation and promoted infection control. Further study is required to demonstrate the influence of the internal exposure dose of smoking, such as nicotine- or tobacco-specific nitrosamine metabolism, and to clarify the mechanism by which smoking and salivary antioxidants affect the quality and effectiveness of periodontal treatment.

## Methods

### Subject recruitment

This is an observational study, and it had no control group. Participants were enrolled from the Department of Periodontology at Taipei Medical University Hospital between July 2013 and April 2016. The periodontal diagnostic criteria were based on the classification of the American Academy of Periodontology^51^ and International Classification of Disease code 523 (ICD-9 523). The code ICD-9 523 was used to diagnose gingival and periodontal diseases, including chronic and aggressive periodontitis. The inclusion criteria were as follows: the patient had been diagnosed with ICD-9 523 and eligible for comprehensive periodontal treatment project (CTPT), which a National Health Insurance (NHI) program is to reduce periodontal disease in Taiwan^52^; and this was their first visit for periodontitis treatment; and the number of functional teeth was >15, the probing depth was ≥5 mm for at least six teeth; and the patient had been treated with non-surgical therapy. Patients who had received periodontal therapy, were pregnant, or had been diagnosed with cancer were excluded from the study. This study was approved by the Research Ethics Committee of Taipei Medical University Joint Institutional Review Board (Taipei, Taiwan) and complied with the World Medical Association’s *Declaration of Helsinki*.

**Figure 3:**
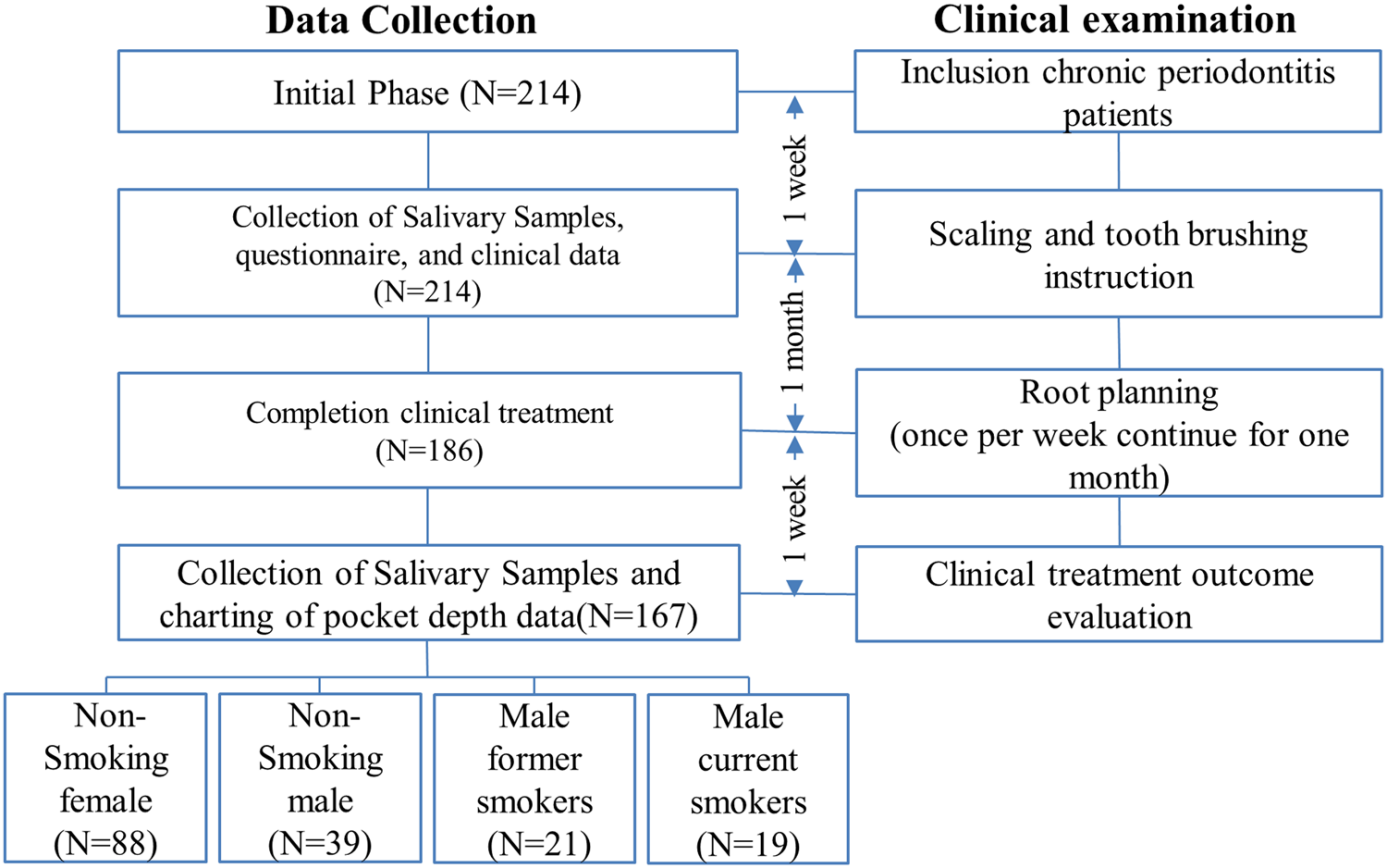
Flowchart of participants’ enrollment.

Figure 3 is a flowchart of participant enrollment. Once patients completed the informed consent form, well-trained interviewers carried out standardized personal interviews based on a structured questionnaire (*N* = 214). The information on the questionnaire included demographics, socioeconomic status, cigarette smoking status (quantity, duration, and pack-years), alcohol consumption, and frequency of betel nut chewing. In total, 186 subjects completed scaling and root planing (once per week for 4 consecutive weeks). The information on the individual and family medical histories, such as inflammatory diseases and cardiovascular diseases, was requested by using the structured questionnaire and medical records. In this study, even though systemic diseases were related to smoking status, no significant differences regarding oxidative stress was found between participants with and without the systemic diseases or with and without medication use. Participants verified their smoking history, including age at start, time since quitting, duration and intensity. A smoker was defined as one who smoked tobacco regularly more than three days per week for a duration exceeding six months. After excluding those with incomplete data on clinical indices, 167 subjects (88 non-smoking females, 39 non-smoking males, 21 former male smokers, and 19 current male smokers) were recruited for this study.

In this study, we planned a study with 167 pairs of saliva samples to measure five salivary oxidative stress markers. If the standard deviation of salivary Cu/Zn SOD, MnSOD, and Catalase was 9.47 or 4.55 or 58822.98 µg/ml and if the true difference in the mean response of matched pairs was 2.97, 2.41, and 15259.69 µg/ml, respectively, we would be able to reject the null hypothesis that this response difference is zero with a power of 0.98, 1.00, and 0.91, respectively. The power for Trx1 and Prx2 was 0.25 and 0.11, respectively.

### Saliva collection and physical properties of saliva

Saliva samples were collected when participants visited our outpatient clinic. Samples were taken at baseline (before non-surgical intervention) and 1 week after completing clinical treatment. Saliva specimens collation and storage were modified from a previous study^10^. Subjects rinsed their mouth with water to remove food residue and waited at least 10 minutes after rinsing to avoid specimen dilution before saliva collection. Stimulated saliva specimens were collected by chewing paraffin wax for 5 min before periodontal treatment, and the pH, flow rate, and buffer capacity were determined using a Saliva-Check kit (GC Corporation, Tokyo, Japan). After collection, samples were stored in an ice bucket and transported to the laboratory immediately. A protease inhibitor cocktail (Roche Applied Science, Mannheim, Germany) was added to saliva samples at a ratio of 1 ml of saliva: 10 µL of protease inhibitor cocktail. After adding the protease inhibitor, the saliva specimen was centrifuged (3000 rpm) for 3 min at room temperature, and the supernatants were stored at −20 °C until analysis of oxidative stress biomarkers.

### Salivary oxidative stress determination

After salivary proteins were measured by the Bradford protein assay^53^, five oxidative stress biomarkers, copper-zinc (Cu/Zn) SOD, manganese (Mn) SOD, catalase, thioredoxin-1 (Trx1), and peroxiredoxin-2 (Prx2), were determined using a MILLIPLEX® MAP Human Oxidative Stress Magnetic Bead Panel kit (Merck Millipore, Darmstadt, Germany). Utilizing internally color-coded microspheres with two fluorescent dyes and by using precise concentrations of these dyes, distinctly colored beads were created and coated with a specific capture antibody. The procedure was as described below. Each sample (35 μL) was diluted to an identical quantity of protein (10 µg) with assay buffer, added to a 96-well plate, mixed with 35 μL of assay buffer and 25 μL of antibody-immobilized beads, and incubated for 2 h at room temperature, followed by incubation with detection antibodies (50 μL) and streptavidin phycoerythrin (50 μL). The mean fluorescence intensity was determined, and the concentrations of five oxidative stress biomarkers were calculated by comparing the fluorescence units to a calibration curve (0.55 µg/ml, 1.65 µg/ml, 4.94 µg/ml, 14.81 µg/ml, 44.44 µg/ml, 133.33 µg/ml, 400 µg/ml). The *R*-squared value for the standard curve was >0.995. The coefficient of variance (CV) for the intra-assay ranged from 3.49% to 8.10%, and the CV for the inter-assay ranged from 1.22% to 12.30%. The extent of change (Δ) of each of the five salivary biomarkers was calculated by subtracting the concentration at baseline from that after completing treatment. To ensure that researchers did not bias the results of the salivary oxidative stress laboratory tests, the assays were performed in a single-blind design.

### Periodontal clinical evaluation

All clinical examinations and non-surgical therapy were carried out by a periodontist. Non-surgical periodontal treatment included subgingival scaling, root planing, and oral hygiene instructions. Initially, each patient received scaling and tooth-brushing instructions. To improve the efficacy of periodontal therapy, smoking subjects were told to stop/reduce cigarette smoking during non-surgical periodontal therapy. Some smokers obeyed the instruction to reduce cigarette smoking when they were under non-surgical periodontal therapy, but none of them quit. Afterwards, root planing was implemented once per week for 4 weeks. Mechanical subgingival instrumentation was performed using hand instruments (Gracey curette No. 1/2, 3/4, 11/12, and 13/14) and a scaling tip. The clinical parameters, such as the plaque index (PI), bleeding on probing (BOP), and probing depth (PD), were collected at baseline and after completing clinical treatment. The PI measurement was based on the plaque control record^54^ and both soft debris and mineralized deposits on the four surfaces (buccal, lingual, mesial, and distal) of each tooth, and the presence or absence of plaque was recorded for all sites. PI was calculated by dividing the number of plaque-containing surfaces by the total number of available surfaces. BOP and PD were measured using a periodontal probe at six sites (distobuccal, buccal, mesiobuccal, distolingual, lingual, and mesiolingual) on each tooth. The PD recovery rate was defined as an improvement in the PD of >2 mm for at least one periodontal pocket of 5–9 mm on each side of the mouth. Periodontal examinations at baseline and after treatment were performed by the same periodontist.

### Statistical analysis

All statistical analyses were performed with SAS 9.4 (SAS Institute, Cary, NC, USA). The association between the demographic characteristics and smoking status were determined by the chi-squared test (categorical variables) and Wilcoxon rank-sum test or Kruskal-Wallis test with Bonferroni post hot test (continuous variables). The median and quartile deviation (QD) of the clinical measurements and salivary marker levels at baseline and after treatment according to the smoking status are presented in a histogram. The Wilcoxon signed-rank test was used to compare the periodontal parameters and salivary antioxidant biomarkers at baseline and after completing clinical treatment. The strength of the correlation between the periodontal parameters and salivary antioxidant biomarkers at baseline or after completing clinical treatment was determined by Spearman’s correlation coefficient. The minimal and maxima data of Cu/Zn SOD, MnSOD, catalase, TRX1and PRX2 in baseline were 0.96–88.3, 0.26–25.65, 1.17–377253.00, 0.20–1001.00, and 0.15–4504.00 µg/ml, respectively. The minimal and maxima data of Cu/Zn SOD, MnSOD, catalase, TRX1and PRX2 after treatment were 0.22–52.76, 0.18–19.49, 0.44–117811.00, 11.45–1015.00 and 0.14–8441.00 µg/ml, respectively. The ranges of data on catalase, TRX1and PRX2 were wildly and were log-transformed for boxplot presentation of Fig. 2. To further explore the interaction between salivary antioxidant biomarkers and smoking on periodontal clinical parameters, two-way repeated-measures ANOVA was performed comparing subject performance according to the clinical parameters before and after treatment, using the smoking status and oxidative stress marker difference as the main effects (independent variables). The effect of smoking status was classified into non-smoking females, non-smoking males, former smoking males, and currently smoking males (four levels). The effects of the differences in the five salivary oxidative stress markers were classified into three levels. The cutoff points distinguishing the three levels were zero and the median of the values that were above zero. For example, 61 subjects had a ΔCu/Zn SOD that was less than zero, and the median of the other subjects’ (N = 106) ΔCu/Zn SOD was 5.4 µg/ml. The cutoff points creating the three levels of ΔCu/Zn SOD were thus 0 and 5.4, meaning the three levels of ΔCu/Zn SOD were <0, 0–5.4 µg/ml, and ≥5.4 µg/ml. The median values of ΔMnSOD, ΔCatalase, ΔTrx1 and ΔPrx2 that were above 0 were 2.5, 225, 185, and 0.6 µg/ml, respectively. If the interactions of smoking status and oxidative stress marker difference were showed significance in clinical parameters, post-hoc with Bonferroni correction after two-way ANOVA with repeated measure was used to test the difference. The level of statistical significance was *p* < 0.05.

## Electronic supplementary material

Supplementary Tables

## References

[1] Petersen PE, Ogawa H (2012). The global burden of periodontal disease: towards integration with chronic disease prevention and control. Periodontol. 2000.

[2] Eke PI (2016). Predicting Periodontitis at State and Local Levels in the United States. J Dent Res.

[3] Eke, P. I., Dye, B. A., Wei, L., Thornton-Evans, G. O. & Genco, R. J. Prevalence of periodontitis in adults in the United States: 2009 and 2010. *J Dent. Res* **91**, 914–920 (2012). 10.1177/0022034512457373 22935673

[4] Department of Health, T. Bureau of Health Promotion Annual Report, 2015 (2015).

[5] Yu HC, Su NY, Huang JY, Lee SS, Chang YC (2017). Trends in the prevalence of periodontitis in Taiwan from 1997 to 2013: A nationwide population-based retrospective study. Medicine (Baltimore).

[6] Hernandez M (2011). Host-pathogen interactions in progressive chronic periodontitis. J. Dent. Res.

[7] Netto LE, Antunes F (2016). The Roles of Peroxiredoxin and Thioredoxin in Hydrogen Peroxide Sensing and in Signal Transduction. Mol Cells.

[8] Droge W (2002). Free radicals in the physiological control of cell function. Physiol Rev.

[9] Sculley DV, Langley-Evans SC (2003). Periodontal disease is associated with lower antioxidant capacity in whole saliva and evidence of increased protein oxidation. Clinical science.

[10] Kim SC, Kim OS, Kim OJ, Kim YJ, Chung HJ (2010). Antioxidant profile of whole saliva after scaling and root planing in periodontal disease. J Periodontal Implant. Sci.

[11] Novakovic N (2013). Antioxidative status of saliva before and after non-surgical periodontal treatment. Srp. Arh. Celok. Lek.

[12] Wei D, Zhang XL, Wang YZ, Yang CX, Chen G (2010). Lipid peroxidation levels, total oxidant status and superoxide dismutase in serum, saliva and gingival crevicular fluid in chronic periodontitis patients before and after periodontal therapy. Aust. Dent J.

[13] Tomar SL, Asma S (2000). Smoking-Attributable Periodontitis in the United States: Findings From NHANES III. Journal of Periodontology.

[14] Eke PI (2016). Risk Indicators for Periodontitis in US Adults: NHANES 2009 to 2012. J Periodontol.

[15] Heasman L (2006). The effect of smoking on periodontal treatment response: a review of clinical evidence. J. Clin. Periodontol.

[16] Goncalves RB (2011). Impact of smoking on inflammation: overview of molecular mechanisms. Inflamm. Res.

[17] Javed F, Bashir AH, Romanos GE (2014). Association between environmental tobacco smoke and periodontal disease: a systematic review. Environ. Res.

[18] Lee J, Taneja V, Vassallo R (2012). Cigarette smoking and inflammation: cellular and molecular mechanisms. J. Dent. Res.

[19] Sanz M, Teughels W (2008). Innovations in non-surgical periodontal therapy: Consensus Report of the Sixth European Workshop on Periodontology. J. Clin. Periodontol.

[20] Caffesse RG, Mota LF, Morrison EC (1995). The rationale for periodontal therapy. Periodontol 2000.

[21] Hughes FJ (2006). Prognostic factors in the treatment of generalized aggressive periodontitis: I. Clinical features and initial outcome. J. Clin. Periodontol.

[22] Knofler GU, Purschwitz RE, Jentsch HF (2007). Clinical evaluation of partial- and full-mouth scaling in the treatment of chronic periodontitis. J Periodontol.

[23] Stenman J, Lundgren J, Wennstrom JL, Ericsson JS, Abrahamsson KH (2012). A single session of motivational interviewing as an additive means to improve adherence in periodontal infection control: a randomized controlled trial. J Clin Periodontol.

[24] Hughes FJ (2006). Prognostic factors in the treatment of generalized aggressive periodontitis: II. Effects of smoking on initial outcome. J Clin Periodontol.

[25] Rosa EF (2011). A prospective 12-month study of the effect of smoking cessation on periodontal clinical parameters. J Clin Periodontol.

[26] Scabbia A, Cho KS, Sigurdsson TJ, Kim CK, Trombelli L (2001). Cigarette smoking negatively affects healing response following flap debridement surgery. J Periodontol.

[27] Preshaw PM, Holliday R, Law H, Heasman PA (2013). Outcomes of non-surgical periodontal treatment by dental hygienists in training: impact of site- and patient-level factors. Int. J. Dent. Hyg.

[28] Papantonopoulos GH (1999). Smoking influences decision making in periodontal therapy: a retrospective clinical study. J. Periodontol.

[29] Mavropoulos A, Aars H, Brodin P (2003). Hyperaemic response to cigarette smoking in healthy gingiva. J Clin Periodontol.

[30] Bergstrom J, Bostrom L (2001). Tobacco smoking and periodontal hemorrhagic responsiveness. J. Clin. Periodontol.

[31] Scheres N, Laine ML, de Vries TJ, Everts V, van Winkelhoff AJ (2010). Gingival and periodontal ligament fibroblasts differ in their inflammatory response to viable Porphyromonas gingivalis. J Periodontal Res.

[32] Waddington RJ, Moseley R, Embery G (2000). Reactive oxygen species: a potential role in the pathogenesis of periodontal diseases. Oral Dis.

[33] Imatani T, Kato T, Okuda K (2001). Production of inflammatory cytokines by human gingival fibroblasts stimulated by cell-surface preparations of Porphyromonas gingivalis. Oral Microbiol Immunol.

[34] Socransky SS, Haffajee AD (1993). Effect of therapy on periodontal infections. J. Periodontol.

[35] van der Weijden GA, Hioe KP (2005). A systematic review of the effectiveness of self-performed mechanical plaque removal in adults with gingivitis using a manual toothbrush. J Clin Periodontol.

[36] Chapple IL (1997). Reactive oxygen species and antioxidants in inflammatory diseases. J Clin Periodontol.

[37] Brock GR, Butterworth CJ, Matthews JB, Chapple IL (2004). Local and systemic total antioxidant capacity in periodontitis and health. J Clin Periodontol.

[38] Novakovic N (2014). Salivary antioxidants as periodontal biomarkers in evaluation of tissue status and treatment outcome. J Periodontal Res.

[39] McCord JM, Edeas MA (2005). SOD, oxidative stress and human pathologies: a brief history and a future vision. Biomed. Pharmacother.

[40] Suzuki K (2003). The relationship between smoking habits and serum levels of 8-OHdG, oxidized LDL antibodies, Mn-SOD and carotenoids in rural Japanese residents. J. Epidemiol.

[41] Bolzan AD, Bianchi MS, Bianchi NO (1997). Superoxide dismutase, catalase and glutathione peroxidase activities in human blood: influence of sex, age and cigarette smoking. Clin. Biochem.

[42] Goth L (1989). Effect of age, sex, and smoking on serum catalase activity. Acta Biol. Hung.

[43] van der Weijden F, Slot DE (2011). Oral hygiene in the prevention of periodontal diseases: the evidence. Periodontol. 2000.

[44] Ooshima T (1993). Oolong tea polyphenols inhibit experimental dental caries in SPF rats infected with mutans streptococci. Caries Res.

[45] Yang PS (2014). Scaling-stimulated salivary antioxidant changes and oral-health behavior in an evaluation of periodontal treatment outcomes. ScientificWorldJournal.

[46] Koseoglu S, Saglam M, Pekbagriyanik T, Savran L, Sutcu R (2015). Level of Interleukin-35 in Gingival Crevicular Fluid, Saliva, and Plasma in Periodontal Disease and Health. J Periodontol.

[47] Lin P-H (2015). Research performance of biomarkers from biofluids in periodontal disease publications. Journal of Dental Sciences.

[48] Giannobile WV (2009). Saliva as a diagnostic tool for periodontal disease: current state and future directions. Periodontol. 2000.

[49] Husain K, Mejia J, Lalla J, Kazim S (2005). Dose response of alcohol-induced changes in BP, nitric oxide and antioxidants in rat plasma. Pharmacol Res.

[50] Budzynski J, Ziolkowski M, Klopocka M, Czarnecki D (2016). Oxidoreductive homeostasis in alcohol-dependent male patients and the risk of alcohol drinking relapse in a 6-month follow-up. Alcohol.

[51] Wiebe CB, Putnins EE (2000). The periodontal disease classification system of the American Academy of Periodontology–an update. J Can. Dent. Assoc.

[52] Chan CL, You HJ, Lian HJ, Huang CH (2016). Patients receiving comprehensive periodontal treatment have better clinical outcomes than patients receiving conventional periodontal treatment. J Formos Med Assoc.

[53] Bradford MM (1976). A rapid and sensitive method for the quantitation of microgram quantities of protein utilizing the principle of protein-dye binding. Anal Biochem.

[54] O’Leary TJ, Drake RB, Naylor JE (1972). The plaque control record. J Periodontol.

