# Supplementary material for: Cigarette Smoking Aggravates the Activity of Periodontal Disease by Disrupting Redox Homeostasis- An Observational Study
Source: Sci Rep. 2018 Jul 23;8:11055. doi: 10.1038/s41598-018-29163-6 (PMC6056417; doi:10.1038/s41598-018-29163-6)
Supplement: Supplementary file 1 — Supplementary Tables [file 41598_2018_29163_MOESM1_ESM.doc]

Supplementary Tables

Cigarette Smoking Aggravates the Activity of Periodontal Disease by Disrupting Redox Homeostasis- An Observational Study

Chia-Huang Chang, PhD^1+^, Ming-Lun Han, MD^2+^, Nai-Chia Teng, DDS, PhD^3^, Chang-Yu Lee DDS, MS^4^, Wan-Ting Huang, MS^3^, Che-Tong Lin DDS, PhD^3^, Yung-Kai Huang, PhD^5*^

^1^ College of Public Health and Nutrition, Taipei Medical University, Taipei, 110, Taiwan

^2^ Department of Internal Medicine, Min-Sheng General Hospital, Taoyuan, 330, Taiwan

^3^ School of Dentistry, College of Oral Medicine, Taipei Medical University, Taipei, 110, Taiwan

^4^ Division of Periodontics, Department of Dentistry, Taipei Medical University Hospital, Taipei Medical University, Taipei, 110, Taiwan

^5^School of Oral Hygiene, College of Oral Medicine, Taipei Medical University, Taipei, 110, Taiwan

^*^ Corresponding author email: [ykhuang@tmu.edu.tw](mailto:ykhuang@tmu.edu.tw)

^+^ These authors contributed equally to this work.

Table S1: Baseline characteristics among non-smokers, former smokers, and current smokers

|  | Female | Male | | | *p* value |
| --- | --- | --- | --- | --- | --- |
|  | Non-smoker | Non-smoker | Former smokers | Current smokers |  |
| Total subject Number | 88 | 39 | 21 | 19 |  |
| Years of schooling, N (%) | |  |  |  |  |
| ≤9 years | 11 (12.50) | 3 (7.69) | 0 (0.00) | 1 (5.26) | 0.54 ^a^ |
| 10~12 years | 26 (29.55) | 13 (33.33) | 7 (33.33) | 9 (47.37) |  |
| ≥13 years | 51 (57.95) | 23 (58.97) | 14 (66.67) | 9 (47.37) |  |
| Marital status, N (%) |  |  |  |  |  |
| Single | 17 (19.32) | 3 (7.69) | 0 (0.00) | 3 (15.79) | 0.06^a^ |
| Married/separated or divorced | 71 (80.68) | 36 (92.31) | 21 (100.00) | 16 (84.21) |  |
| Salivary properties, Median (QD) | | | | | |
| pH | 7.60 (0.20) | 7.60 (0.20) | 7.60 (0.20) | 7.6 (0.10) | 0.70^b^ |
| Flow rate | 4.00 (0.75) | 5.00 (1.75) | 5.00 (1.00) | 5.00 (2.25) | 0.44^b^ |
| Buffer capacity | 11.00 (1.50) | 12.00 (1.00) | 12.00 (1.00) | 10.00 (3.00) | 0.37 ^b^ |
| Smoking habits, Median (QD) | | | | | |
| Cigarettes/day | | | 10.00 (8.50) | 10.00 (7.50) | 0.61^c^ |
| Duration of smoking (years) | | | 26.78 (11.95) | 31.82 (5.70) | 0.29^c^ |
| Total pack-years | | | 18.52 (14.10) | 19.23 (16.13) | 0.46^c^ |

QD, quartile deviation.

^a^: *p* value for Chi-squared; ^b^ *p* value for Kruskal-Wallis test; ^c^: *p* value for Wilcoxon rank-sum test

Table S2: Clinical parameters at baseline and after periodontal treatment strata by smoking status

| Clinical parameters |  | At the baseline | | After completing treatment | | p value^a^ |
| --- | --- | --- | --- | --- | --- | --- |
|  |  | Median | Q1-Q3 | Median | Q1-Q3 |  |
| Plaque index (%) | | | | | | |
| Total | 167 | 62.20 | 45.10-74.70 | 37.30 | 25.00-48.70 | <0.01 |
| Non-smoking females | 88 | 60.40 | 48.50-75.00 | 36.90 | 24.70-47.60 | <0.01 |
| Non-smoking males | 39 | 62.50 | 43.00-76.30 | 40.10 | 26.00-54.50 | <0.01 |
| Male former smokers | 21 | 67.30 | 48.90-73.00 | 40.90 | 27.00-44.6.0 | <0.01 |
| Male current smokers | 19 | 53.00 | 40.00-71.40 | 39.70 | 22.20-48.00 | <0.01 |
| Bleeding on probing (%) | | | | | | |
| Total | 167 | 42.00 | 28.16-55.56 | 18.59 | 11.72-30.55 | <0.01 |
| Non-smoking females | 88 | 42.22 | 28.84-55.28 | 17.58 | 11.72-31.35 | <0.01 |
| Non-smoking males | 39 | 46.66 | 38.69-64.36 | 21.33 | 14.49-32.00 | <0.01 |
| Male former smokers | 21 | 36.51 | 24.69-58.97 | 15.59 | 9.26-29.17 | <0.01 |
| Male current smokers | 19 | 29.31 | 17.28-35.9 | 19.14 | 11.72-31.55 | <0.01 |
| Mean of probing depth (mm) | | | | | | |
| Total | 167 | 3.35 | 3.11-3.69 | 2.71 | 2.58-2.97 | <0.01 |
| Non-smoking females | 88 | 3.35 | 3.11-3.60 | 2.69 | 2.57-2.93 | <0.01 |
| Non-smoking males | 39 | 3.38 | 3.04-3.96 | 2.74 | 2.56-3.10 | <0.01 |
| Male former smokers | 21 | 3.40 | 3.15-3.67 | 2.81 | 2.67-2.96 | <0.01 |
| Male current smokers | 19 | 3.20 | 3.07-3.53 | 2.67 | 2.60-2.78 | <0.01 |

^a^ Wilcoxon signed-rank test

Table S3: Reduction of clinical parameters strata by smoking status

|  | Females | Males | | | p-value^a^ |
| --- | --- | --- | --- | --- | --- |
| Clinical parameters | Non-smoker | Non-smoker | Former smokers | Current smokers |  |
|  | Median (Q1-Q3) | | | |  |
| Plaque index (%) | 23.65  (7.75-35.45) | 22.00  (1.35-37.8) | 26.80  (13.5-40.1) | 16.10  (1.20-28.5) | 0.35 |
| Bleeding on probing (%) | 21.33  (10.37-36.79) | 27.34  (9.17-38.88) | 23.81  (6.43-30.95) | 6.84  (-0.59-20.83) | <0.01 |
| Mean of probing depth (mm) | 0.62  (0.48-0.82) | 0.71  (0.41-1.12) | 0.65  (0.37-0.83) | 0.57  (0.3-0.75) | 0.37 |
| PD recovery rate (%) | 92.65  (87.25-100.00) | 92.8  (82-100.00) | 88.20  (80.00-95.4) | 91.00  (85.70-100.00) | 0.51 |

^a^Kruskal-Wallis test

Table S4: Salivary biomarkers at baseline and after periodontal treatment strata by smoking status

| Salivary biomarkers | N | At the baseline | | After completing treatment | | p value^a^ |
| --- | --- | --- | --- | --- | --- | --- |
|  |  | Median | Q1-Q3 | Median | Q1-Q3 |  |
| Cu/Zn SOD (µg/ml) | | | | | | |
| Total | 167 | 10.51 | 6.5-16.14 | 8.55 | 4.77-13.13 | <0.0001 |
| Non-smoking females | 88 | 11.45 | 7.65-16.56 | 8.55 | 5.76-13.6 | <0.01 |
| Non-smoking males | 39 | 10.51 | 5.48-21.98 | 8.41 | 4.02-13.13 | <0.01 |
| Male former smokers | 21 | 8.41 | 5.38-16.14 | 9.16 | 4.61-13.83 | 0.40 |
| Male current smokers | 19 | 7.46 | 4.38-11.85 | 7.20 | 3.27-9.98 | 0.99 |
| Mn SOD(µg/ml) | | | | | | |
| Total | 167 | 4.31 | 2.25-7.46 | 2.58 | 1.43-4.61 | <0.0001 |
| Non-smoking females | 88 | 4.09 | 2.51-7.21 | 3.01 | 1.61-4.69 | <0.0001 |
| Non-smoking males | 39 | 5.03 | 2.49-6.96 | 2.49 | 1.32-4.61 | <0.0001 |
| Male former smokers | 21 | 5.67 | 1.05-8.55 | 1.86 | 1.13-5.29 | <0.01 |
| Male current smokers | 19 | 3.27 | 1.83-7.46 | 2.10 | 1.35-3.51 | 0.07 |
| Catalase (µg/ml) | | | | | | |
| Total | 167 | 247.35 | 58.49-845.46 | 94.58 | 36.17-287.13 | <0.0001 |
| Non-smoking females | 88 | 247.35 | 76.98-2727.5 | 138.06 | 46.1-318.05 | <0.0001 |
| Non-smoking males | 39 | 287.13 | 62.65-456.84 | 54.61 | 23.14-237.92 | <0.0001 |
| Male former smokers | 21 | 287.13 | 40.09-874.46 | 108.51 | 38.08-180.05 | 0.03 |
| Male current smokers | 19 | 116.22 | 42.94-444.25 | 67.10 | 26.1-180.05 | 0.01 |
| Log of Catalase (µg/ml) |  |  |  |  |  |  |
| Total | 167 | 2.39 | 1.77-2.93 | 1.98 | 1.56-2.46 | <0.0001 |
| Non-smoking females | 88 | 2.39 | 1.89-3.39 | 2.14 | 1.66-2.5 | <0.0001 |
| Non-smoking males | 39 | 2.46 | 1.8-2.66 | 1.74 | 1.36-2.38 | <0.0001 |
| Male former smokers | 21 | 2.46 | 1.6-2.94 | 2.04 | 1.58-2.26 | 0.04 |
| Male current smokers | 19 | 2.07 | 1.63-2.65 | 1.83 | 1.42-2.26 | 0.02 |
| TRX1 (µg/ml) |  |  |  |  |  |  |
| Total | 167 | 589.69 | 154.11-768.43 | 516.27 | 124.48-805.34 | 0.04 |
| Non-smoking females | 88 | 609.33 | 215.56-775.03 | 557.12 | 173.07-805.39 | 0.23 |
| Non-smoking males | 39 | 568.26 | 166.42-768.43 | 166.42 | 64.83-820.32 | 0.06 |
| Male former smokers | 21 | 328.34 | 91.39-701.13 | 585.77 | 101.31-672.8 | 0.92 |
| Male current smokers | 19 | 650.91 | 85.33-836.63 | 516.58 | 94.58-819.41 | 0.89 |
| Log of TRX1 (µg/ml) |  |  |  |  |  |  |
| Total | 167 | 2.77 | 2.19-2.89 | 2.71 | 2.1-2.91 | 0.01 |
| Non-smoking females | 88 | 2.78 | 2.33-2.89 | 2.75 | 2.24-2.91 | 0.15 |
| Non-smoking males | 39 | 2.75 | 2.22-2.89 | 2.22 | 1.81-2.91 | 0.01 |
| Male former smokers | 21 | 2.52 | 1.96-2.85 | 2.77 | 2.01-2.83 | 0.86 |
| Male current smokers | 19 | 2.81 | 1.93-2.92 | 2.71 | 1.98-2.91 | 0.56 |
| PRX2 (µg/ml) |  |  |  |  |  |  |
| Total | 167 | 1.15 | 0.84-2.58 | 1.06 | 0.79-2.17 | 0.14 |
| Non-smoking females | 88 | 1.14 | 0.87-1.91 | 1.11 | 0.80-2.14 | 0.70 |
| Non-smoking males | 39 | 1.35 | 0.75-5.29 | 0.98 | 0.77-2.41 | <0.001 |
| Male former smokers | 21 | 1.22 | 1.00-2.95 | 1.25 | 0.92-2.36 | 0.88 |
| Male current smokers | 19 | 1.00 | 0.79-1.43 | 0.89 | 0.74-1.96 | 0.36 |
| Log of PRX2 (µg/ml) |  |  |  |  |  |  |
| Total | 167 | 0.06 | -0.08-0.41 | 0.03 | -0.1-0.34 | 0.17 |
| Non-smoking females | 88 | 0.06 | -0.06-0.28 | 0.04 | -0.1-0.33 | 0.80 |
| Non-smoking males | 39 | 0.13 | -0.12-0.72 | -0.01 | -0.11-0.38 | <0.01 |
| Male former smokers | 21 | 0.09 | 0.00-0.47 | 0.10 | -0.04-0.37 | 0.85 |
| Male current smokers | 19 | 0.00 | -0.1-0.16 | -0.05 | -0.13-0.29 | 0.52 |

^a^Wilcoxon signed-rank test
